# Non-secreting pituitary tumours characterised by enhanced expression of YAP/TAZ

**DOI:** 10.1530/ERC-18-0330

**Published:** 2018-08-21

**Authors:** Paraskevi Xekouki, Emily J Lodge, Jakob Matschke, Alice Santambrogio, John R Apps, Ariane Sharif, Thomas S Jacques, Simon Aylwin, Vincent Prevot, Ran Li, Jörg Flitsch, Stefan R Bornstein, Marily Theodoropoulou, Cynthia L Andoniadou

**Affiliations:** 1 Centre for Craniofacial and Regenerative BiologyKing’s College London, London, UK; 2 Department of EndocrinologyKing’s College Hospital NHS Foundation Trust, London, UK; 3 Department of Endocrinology and DiabetesKing’s College London, London, UK; 4 Institute of NeuropathologyUniversity Hospital Hamburg-Eppendorf, Hamburg, Germany; 5 Department of Internal Medicine IIICarl Gustav Carus Medical School, Technical University of Dresden, Dresden, Germany; 6 Birth Defects Research CentreDevelopmental Biology and Cancer Programme, UCL Great Ormond Street Institute of Child Health, University College London, London, UK; 7 Histopathology DepartmentGreat Ormond Street Hospital NHS Trust, London, UK; 8 Laboratory of Development and Plasticity of the Neuroendocrine BrainInserm U1172, Jean-Pierre Aubert Research Centre, Lille, France; 9 Department of NeurosurgeryTongji Hospital, Tongji Medical College, Huazhong University of Science and Technology, Wuhan, People’s Republic of China; 10 Department of NeurosurgeryHamburg University Medical Center, Hamburg, Germany; 11 Medizinische Klinik und Poliklinik IVLudwig-Maximilians-Universität München, Munich, Germany

**Keywords:** Hippo signalling, pituitary tumour, YAP, TAZ

## Abstract

Tumours of the anterior pituitary can manifest from all endocrine cell types but the mechanisms for determining their specification are not known. The Hippo kinase cascade is a crucial signalling pathway regulating growth and cell fate in numerous organs. There is mounting evidence implicating this in tumour formation, where it is emerging as an anti-cancer target. We previously demonstrated activity of the Hippo kinase cascade in the mouse pituitary and nuclear association of its effectors YAP/TAZ with SOX2-expressing pituitary stem cells. Here, we sought to investigate whether these components are expressed in the human pituitary and if they are deregulated in human pituitary tumours. Analysis of pathway components by immunofluorescence reveals pathway activity during normal human pituitary development and in the adult gland. Poorly differentiated pituitary tumours (null-cell adenomas, adamantinomatous craniopharyngiomas (ACPs) and papillary craniopharyngiomas (PCPs)), displayed enhanced expression of pathway effectors YAP/TAZ. In contrast, differentiated adenomas displayed lower or absent levels. Knockdown of the kinase-encoding *Lats1* in GH3 rat mammosomatotropinoma cells suppressed *Prl* and *Gh* promoter activity following an increase in YAP/TAZ levels. In conclusion, we have demonstrated activity of the Hippo kinase cascade in the human pituitary and association of high YAP/TAZ with repression of the differentiated state both *in vitro* and *in vivo*. Characterisation of this pathway in pituitary tumours is of potential prognostic value, opening up putative avenues for treatments.

## Introduction

The Hippo kinase cascade is a crucial conserved signalling pathway regulating organ growth across diverse species, through the control of cell proliferation, apoptosis and differentiation during development (Zhao *et al.* 2011). This phosphorylation cascade in mammals includes core kinases MST1/2 (a.k.a. STK3/4, orthologues of *Drosophila* Hippo) that activate large tumour suppressor homologs 1 and 2 (LATS1/2), which in turn phosphorylate and inhibit the activation of transcriptional co-activators Yes-associated protein (YAP), and WW domain-containing transcription factor (TAZ/WWTR1), the major effectors of the cascade. YAP/TAZ act as co-activators to TEAD transcription factors (TEA domain family members 1–4) (Zhao *et al.* 2011). Nuclear YAP/TAZ are thus associated with low kinase activity and the promotion of growth, inhibition of apoptosis and the stem cell state, whilst cytoplasmic and phosphorylated YAP/TAZ are associated with active kinases and growth restriction. Deregulation of the mammalian Hippo signalling components has been implicated in the formation of tumours and cancers, with loss of MST1/2, LATS1/2, SAV or MOB1 resulting in the development of different tumour types in mouse models (Mo *et al.* 2014). Furthermore, elevated levels and nuclear localisation of YAP and/or TAZ have been reported in a wide array of human cancers including hepatocellular carcinoma, prostate cancer, colorectal carcinoma (CRC), non-small-cell lung cancer (NSCLC), ovarian cancer, clear cell renal cell carcinoma (ccRCC), pancreatic carcinoma, oesophageal squamous cell carcinoma, urothelial carcinoma of the bladder and skin basal cell carcinoma (Zanconato *et al.* 2016).

Pituitary tumours account for 10–15% of intracranial neoplasms (Molitch 2017). Although in general they are considered benign tumours, an aggressive or invasive behaviour is not uncommon (Di Ieva *et al.* 2014). Hormonal secreting (functioning) pituitary tumours are commonly detected due to the clinical syndromes caused by hormonal hypersecretion (Chanson *et al.* 2015). Non-functioning subtypes are clinically challenging because they present at a later stage with local mass effects or hypopituitarism, as do craniopharyngiomas, both the adamantinomatous (ACP) and papillary (PCP) types. During the last years, progress has been made on the identification of mechanisms involved in anterior pituitary cell transformation and tumourigenesis; oncogene activation, tumour suppressor gene inactivation, epigenetic changes and microRNA deregulation have all been shown to contribute to the initiation of pituitary tumours (Marques & Korbonits 2017). Recently, the isolation of cell subpopulations with stem-like characteristics was reported from human somatotropinomas and non-functioning pituitary adenomas, describing expression of stem cell markers (OCT4, SOX2, CD133, Nestin), sustained proliferation and a persistent undifferentiated compartment (Würth *et al.* 2017). There is evidence for a role of the Hippo signalling cascade in pituitary gland pathophysiology. Mice deficient for Lats1 (*Lats1^−^^/^^−^*) present with hyperplasia of the anterior pituitary lobe, but reduced secretion of hormones such as LH, PRL and GH (St John *et al.* 1999). We recently mapped for the first time the activity of the Hippo-YAP/TAZ pathway in the murine pituitary during development and postnatal stages and revealed an association of active Hippo effectors (i.e. nuclear YAP/TAZ localisation) with the uncommitted pituitary stem cells expressing SOX2 (Lodge *et al.* 2016).

Considering the involvement of the Hippo kinase cascade in tumourigenesis, our observations in the murine gland prompted us to examine the expression patterns of YAP and TAZ in normal human pituitary and in pituitary tumours. Herein, we show for the first time that these proteins are expressed during human pituitary development as well as in the normal adult gland. YAP and TAZ were highly expressed in subsets of non-secreting pituitary tumours (null cell, ACP and PCP), but not in differentiated tumours. Activating this pathway *in vitro* by knocking down *Lats1*, decreased anterior pituitary hormone synthesis, further supporting a role for this cascade in repressing endocrine differentiation. Altogether, our data indicate a previously unappreciated involvement of the Hippo pathway in human pituitary differentiation, growth and tumour formation.

## Materials and methods

### Tissue specimens

All procedures performed were in accordance with the ethical standards of the institutional research committee (King’s College Research Ethics Committee, approval number LRS-15/16-2126) and with the 1964 Helsinki declaration and its later amendments or comparable ethical standards. Anonymised archival FFPE specimens of five ACP (Andoniadou *et al.* 2012) and six PCP (Haston *et al.* 2017) were identified in the local pathology archive, through the Childhood’s Cancer and Leukaemia Group Tissue Bank and BRAIN UK. The study also included ten null-cell pituitary tumours, 16 prolactinomas, one PRL-secreting carcinoma (Winkelmann *et al.* 2002), 18 corticotropinomas and 10 somatotropinomas. Examples of confirmatory testing for these cohorts are provided in Supplementary Fig. 2 (see section on supplementary data given at the end of this article). Foetal pituitary tissue (two samples at 17 weeks and one sample at 14 weeks corrected gestational age) was accessed through the Human Developmental Biology Resource. Normal human pituitary glands were obtained from the School of Medicine, Lille, France from people who donated their body to science (two male, two female, ages 77–89 years old); permission to use human tissues was obtained from the French Agency for Biomedical Research (Agence de la Biomédecine, Saint-Denis la Plaine, France, protocol no. PFS16-002). Ischaemia time until fixation ranged between 8 and 39 h. Pituitaries were fixed in 4% paraformaldehyde at 4°C for a minimum of 48 h before processing for paraffin embedding.

### Immunofluorescence

Samples were dewaxed in Histo-Clear (National Diagnostics) twice for 10 min, followed by rehydration through a descending ethanol series. Antigen retrieval was carried out in citrate-based Declere unmasking solution (Cell Marque) in a Decloaking chamber NXGEN (Menarini Diagnostics) using the 110°C antigen retrieval protocol. Following blocking for 1 h in TNB-blocking buffer (0.1 M Tris–HCl pH7.5, 0.15 M NaCl, 0.5% Blocking Reagent (FP1020, Perkin Elmer)), samples were incubated overnight in primary antibodies at 4°C in TNB at the following dilutions: YAP (Cell Signaling Technology Cat. No. 4912, 1:1000), pYAP (S127) (Cell Signaling Technology Cat. No. 4911 1:1000), TAZ (Atlas Antibodies HPA007415, 1:2000) and SOX2 (Abcam ab97959, 1:2000). The following day, slides were washed in Tris–NaCl–Tween (TNT) buffer (0.1 M Tris–HCl, pH7.5, 0.15 M NaCl, 0.05% Tween-20) and incubated in species-specific biotinylated secondary antibodies (1:500, Abcam) diluted in TNB for 1 h at room temperature. Following washes in TNT, slides were incubated in ABC solution (Vector Laboratories PK-6100) for 30 min in the dark and in TSA-Cy3 diluted in TSA Stock Solution (Perkin Elmer NEL760001) for 10 min at room temperature. Subsequently, slides were washed and incubated in Hoechst labelling solution for 30 min at room temperature. After a final wash, they were mounted with soft-set mounting medium (Vector Laboratories, H1000). Immunofluorescence staining was assessed as follows: Type A: high levels of both YAP/TAZ, frequent nuclear staining; Type B: robust levels of TAZ with frequent nuclear staining and moderate levels of YAP with occasional nuclear staining; Type C: moderate levels of YAP/TAZ, predominantly cytoplasmic, in over 50% of the tumour; Type D: low levels of YAP/TAZ, predominantly cytoplasmic, between 20 and 50% of tumour; Type E: absent YAP/TAZ staining or low levels in under 20% of the tumour.

### Cell culture, transfection and luciferase assays

GH3 cells (American Type Culture Collection) were cultured in 10% foetal calf serum Dulbecco’s modified Eagle medium (DMEM) supplemented with 2.2 g/L NaHCO_3_, 10 mM HEPES, 2 nM Glutamine and 10^5^ U/L penicillin-streptomycin. Cell culture materials were from Life Technologies, Nunc (Wiesbaden, Germany) and Sigma-Aldrich. Cells were transfected with SuperFect (Qiagen) following the manufacturer’s instructions. siRNA were against rat *Lats1* (OriGene and Santa Cruz Biotechnology); a mix of scrambled non-specific siRNA was used as control. The *Gh* and *Prl* promoter reporter vectors have the proximal (_593) rat *Gh* promoter and rat *Prl* promoter respectively upstream to the luciferase gene (both kind gifts of A. Gutierrez-Hartmann, University of Colorado, Denver, CO, USA). After transfection, cells were left for 48 h in low serum (2% FCS) DMEM, before being treated and/or assayed. The transfection efficacy was determined by cotransfection with the RSV-β-gal construct and results are presented as luciferase:β-galactosidase activity ratio. Each experiment was done in triplicate.

Cell proliferation was determined 48 h after transfection using the WST-1 colorimetric assay (Roche Molecular Biochemicals) following the manufacturer’s instructions.

### Immunoblotting

GH3 cells were lysed in ice-cold RIPA lysis buffer supplemented with protease and phosphatase inhibitor cocktail (Roche). Proteins were separated by polyacrylamide gel electrophoresis and blotted using standard procedures (BioRad). Primary antibodies were against LATS1 (C66B5, #3477), YAP/TAZ (D24E4, #8418) and phosphorylated pYAP(S127) (D9W2I, #13008) (all rabbit mAb, Cell Signaling) and β-actin (mouse mAb, Chemicon). Anti-rabbit or anti-mouse horseradish peroxidase-conjugated secondary antibodies were used (Cell Signaling) and signal was developed with enhanced chemiluminescent solution (Roche). Each experiment was carried out in duplicate.

## Results

### YAP and TAZ are expressed in human foetal and adult pituitaries

**Figure 1:**
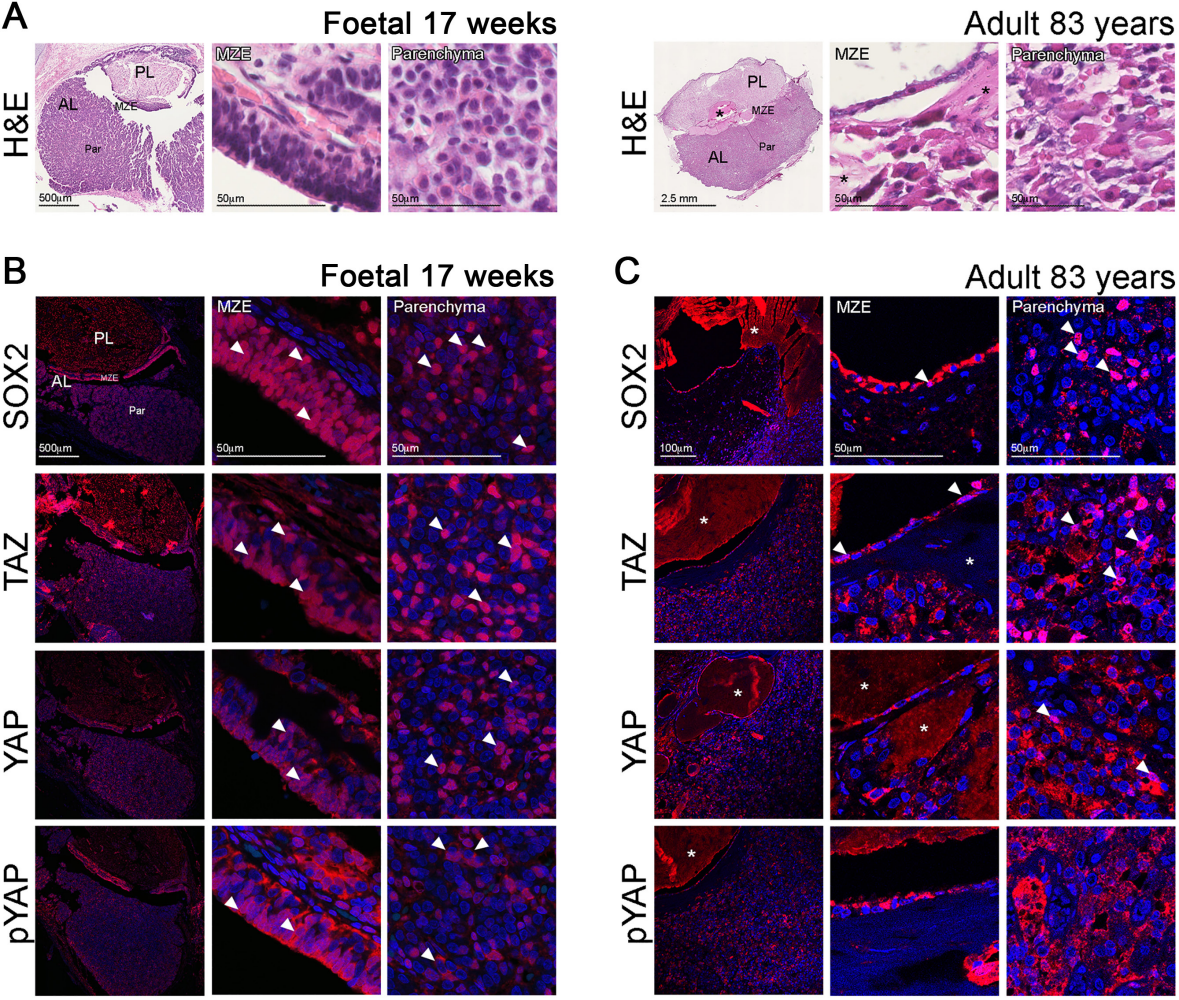
YAP and TAZ are expressed in the human pituitary. (A) Haematoxylin and Eosin staining of sequential frontal sections of human foetal and adult pituitaries. Asterisks denote cysts between the anterior and posterior pituitary. (B) Frontal sections of foetal pituitaries at 17 weeks were stained for SOX2 (pituitary stem/progenitor marker), total TAZ, total YAP and pYAP(S127) marking inactive YAP. Arrowheads indicate examples of cells with nuclear protein. (C) Localisation of SOX2, TAZ, YAP and pYAP proteins in the adult anterior pituitary. Arrowheads indicate examples of cells with nuclear protein, and examples of cytoplasmic localisation for pYAP in (B). AL, anterior lobe; MZE, marginal zone epithelium; Par, parenchyma; PL, posterior lobe.

To investigate the Hippo pathway activity in the human pituitary during development and adulthood, we determined the expression of its downstream effectors YAP and TAZ. In the human foetal pituitary at 17 weeks, high expression of both was observed in the posterior and anterior lobe within the epithelial remnants of Rathke’s pouch (marginal zone epithelium, MZE) that highly express the stem cell marker SOX2; the main body of the anterior lobe presented with moderate YAP and TAZ immunoreactivity (Fig. 1B). Both proteins mainly localised in the nucleus as well as the cytoplasm (arrowheads). The nuclear localisation was more prominent in the case of TAZ (arrowheads) throughout the MZE, similar to SOX2 expression. Nuclear immunoreactivity in the MZE was also observed for YAP, albeit to a lesser extent. The core Hippo kinases LATS1/2 phosphorylate YAP on serines S61, S109, S127, S164 and S381, leading to its inactivation through cytoplasmic retention (S127) and degradation (Zhao *et al.* 2007, 2010). To determine if the kinases are active, we performed immunostaining against pYAP(S127) and observed strong cytoplasmic as well as nuclear localisation in both the epithelium and parenchyma (arrowheads), suggesting that LATS kinases are active during embryonic development. These observations were confirmed in further foetal pituitary samples at 17 and 14 weeks of gestation (Supplementary Fig. 1). To determine if expression of YAP and TAZ persists in the adult pituitary, we analysed immunoreactivities in adult pituitaries of advanced age (*n* = 4) (Fig. 1C). Strong nuclear TAZ staining persisted both in cells of the MZE and parenchyma, following the expression pattern of SOX2. YAP immunoreactivity was predominantly cytoplasmic in the MZE, whilst nuclear staining was occasionally observed in the parenchyma. Staining against pYAP(S127) persisted in the adult pituitary indicating the presence of an active Hippo cascade during adulthood. In summary, YAP and TAZ are expressed in the developing and adult human pituitary, where LATS kinases are active.

### Expression of YAP and TAZ in pituitary tumours

**Figure 2:**
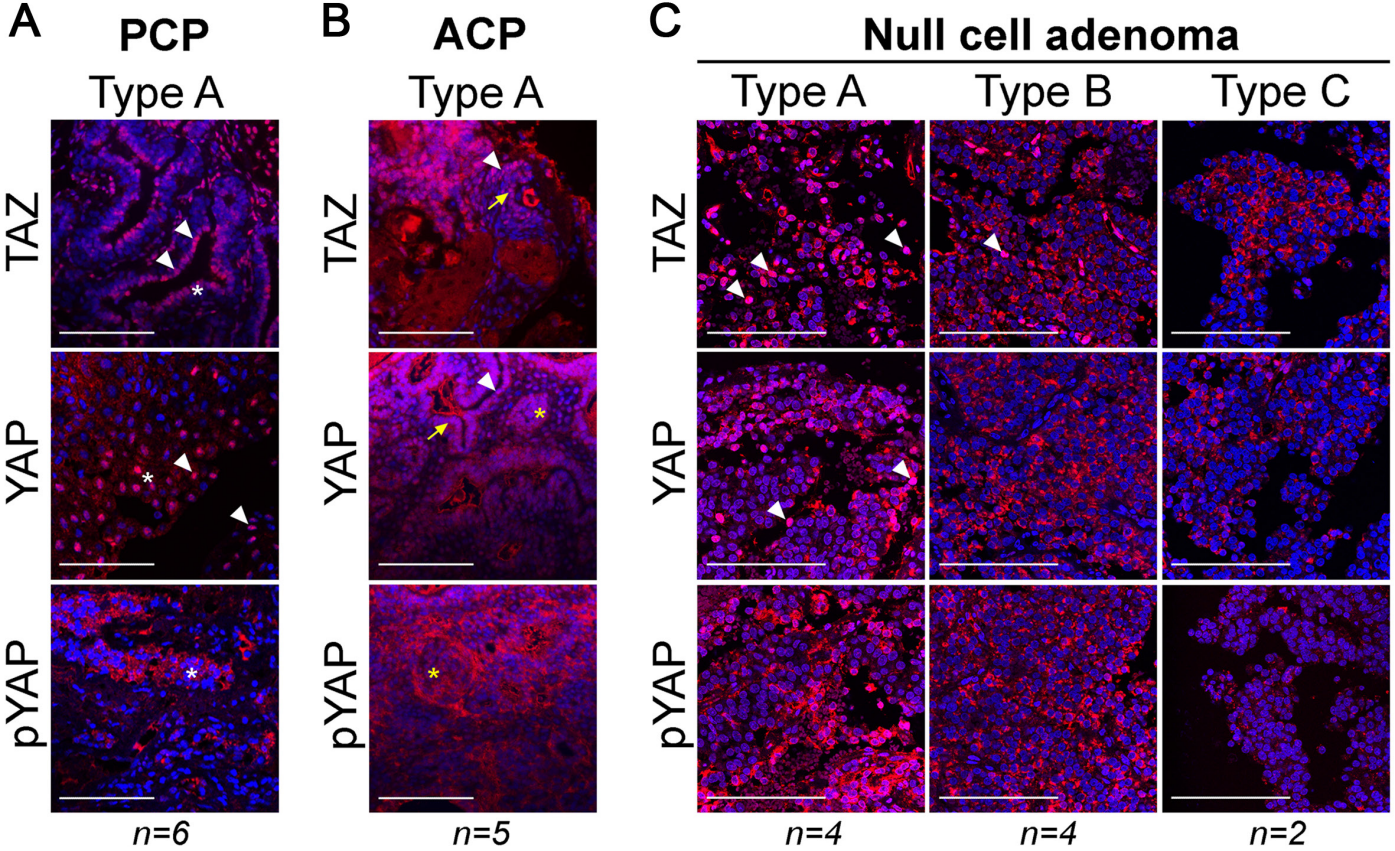
Expression of YAP and TAZ in non-secreting pituitary tumours. (A) Representative immunostaining against TAZ, YAP and pYAP(S127) in samples of papillary craniopharyngioma (PCP). Arrowheads denote examples of nuclear staining; asterisks, the suprabasal squamous epithelium. (B) Representative immunostaining against TAZ, YAP and pYAP(S127) in samples of adamantinomatous craniopharyngioma (ACP). Arrowheads indicate nuclear staining; arrows, the palisading epithelium; asterisks, characteristic whorl-like cluster cells. (C) Immunostaining against TAZ, YAP and pYAP(127) on null cell pituitary adenomas, showing representative staining from tumours classified as Type A, B and C. Arrowheads indicate nuclear staining. Scale bars 100 µm.

We next sought to determine the expression patterns of YAP and TAZ in human pituitary tumours. In human craniopharyngiomas, which are composed mainly of non-endocrine cells, the expression of SOX2/SOX9 (both progenitor/stem cell markers) has been well documented in both the papillary type (PCP), harbouring MAPK pathway mutations as well as the adamantinomatous type (ACP) that harbour *CTNNB1* mutations (encoding β-catenin) (Hölsken *et al.* 2014, Thimsen *et al.* 2017, Haston *et al.* 2017). Strong YAP and TAZ stainings were observed in all PCPs tested and were predominantly nuclear in the basal cells and suprabasal squamous epithelium (asterisks in Fig. 2A), described to robustly express SOX2 (Haston *et al.* 2017) (Fig. 2A and Table 1). Strong nuclear staining for YAP and TAZ was observed in all five ACPs, both in whorl-like formations described to accumulate β-catenin (asterisks in Fig. 2B) as well as in the palisading epithelium (arrows in Fig. 2B). Null-cell pituitary tumours do not show immunoreactivity for any of the pituitary hormones, although there is evidence that in their majority, they express lineage-specific transcription factors (Mete & Lopes 2017, Nishioka & Inoshita 2018). YAP and TAZ expression was variable and was subdivided into three different groups based on a semiquantitative scoring system as described in the methods (Fig. 2C, Table 1). Four out of ten null-cell tumours displayed high immunoreactivity consistent with Type A, three were identified as Type B and three as Type C. There was no obvious correlation of the immunohistochemical data to age at presentation, rate of recurrence, Ki-67 index or p53 levels, although the study cohort is low for this to be accurately determined (Table 2).

**Table 1:** Classification of YAP and TAZ staining in pituitary tumours.

Tumour type	Classification	Number	TAZ	YAP
Normal pituitary	Type B	4	+++ n c	++ n c
PCP	Type A	6	++++ n c	++++ or +++ n c
ACP	Type A	5	++++ n c	++++ or +++ n c
Null-cell adenoma	Type A	4	++++ n c	++++ or +++ n c
	Type B	4	+++ n c	++ n c
	Type C	2	++ or + c	++ or + c
Corticotropinoma	Type B	5	+++ n c	++ n c
	Type C	7	++ or + c	++ or + c
	Type D	6	+ c	+ c
Somatotropinoma	Type B	2	+++ n c	++ n c
	Type C	2	++ or + c	++ or + c
	Type D	5	+ c	+ c
	Type E	1	−	−
Prolactinoma	Type D	11	+ c	+ c
	Type E	5	−	−

−, negative staining; +, positive staining; ACP, adamantinomatous craniopharyngioma; c, cytoplasmic; n, nuclear; PCP, papillary craniopharyngioma.

**Table 2:** Related data for null-cell pituitary adenomas.

Sample	Type	Histology	Ki-67	Notable characteristics	p53 status	Age at presentation	Sex
Null1	A	Null-cell adenoma	2–3%		–	46	F
Null2	A	Null-cell adenoma	3–5%		2–3%	27	F
Null3	A	Null-cell adenoma	3–5%		>1%	48	F
Null4	A	Null-cell adenoma	<3%		–	36	F
Null5	B	Null-cell adenoma	5–7%	recurrence	<1%	41	F
Null6	B	Null-cell adenoma	2–3%		–	73	F
Null7	B	Null-cell adenoma	2–3%		–	31	F
Null8	B	Null-cell adenoma	1–2%		–	49	F
Null9	C	Null-cell adenoma	<2%	Necrotic areas	–	48	M
Null10	C	Null-cell adenoma	2.5%		–	33	F

**Figure 3:**
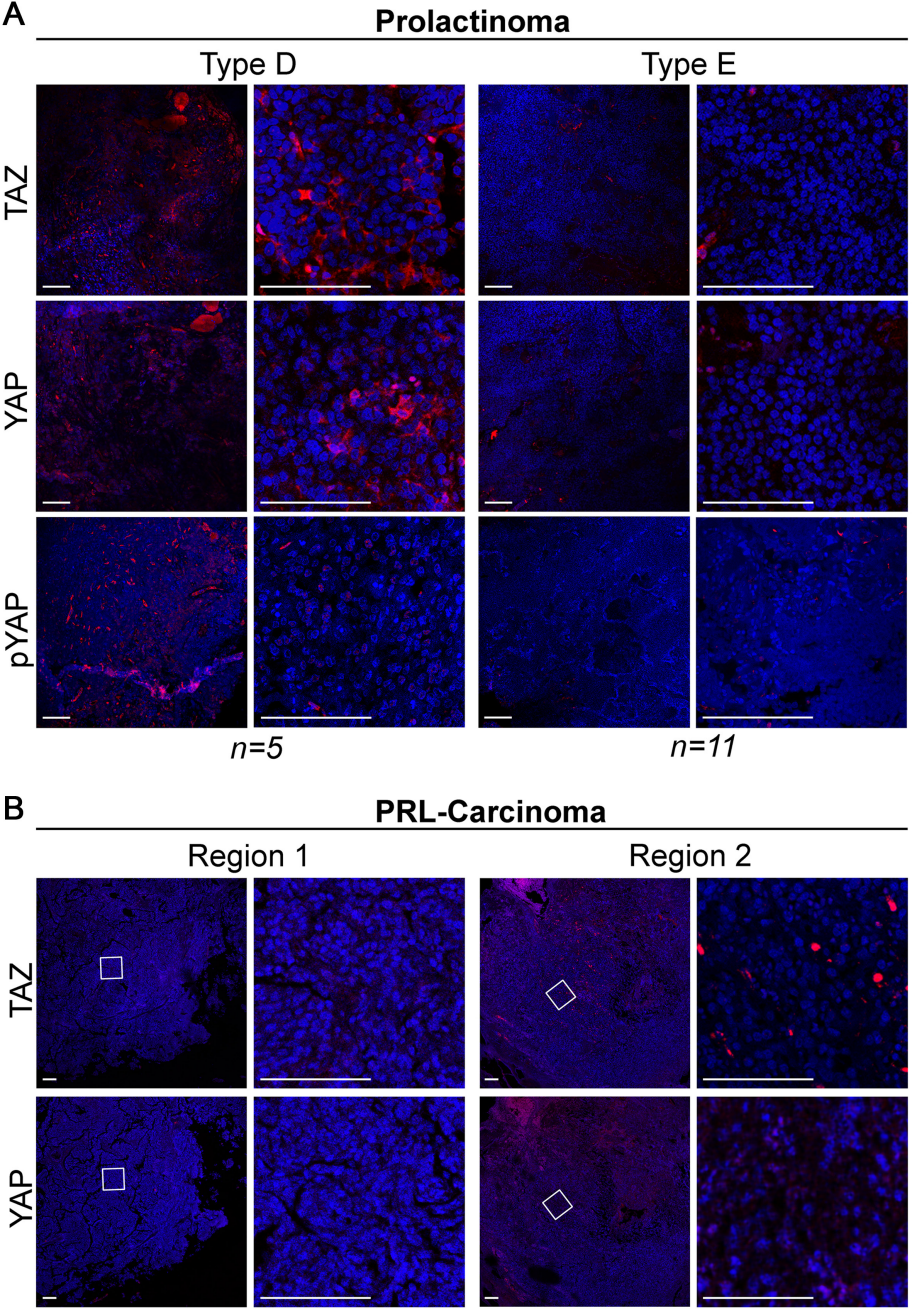
Expression of YAP and TAZ in prolactin-secreting pituitary tumours. (A) Representative immunostaining against TAZ, YAP and pYAP(S127) in prolactinoma samples. Based on the staining patterns tumours were classified as Type D or Type E. (B) Expression of TAZ and YAP in two regions of a prolactin-secreting carcinoma. Scale bars 100 µm.

Altogether our data from normal pituitaries and pituitary tumours suggest that high levels of nuclear YAP/TAZ are associated with a more uncommitted state in the anterior pituitary, consistent with previous mouse data (Lodge *et al.* 2016). To explore this hypothesis further, we extended our study to include 16 prolactinomas, the most common type of secreting pituitary tumours, 10 somatotropinomas and 18 corticotropinomas. YAP and TAZ immunoreactivities were observed at much lower levels than in craniopharyngiomas and null-cell pituitary adenomas. Based on our semiquantitative scoring system, two types of staining were detected in prolactinomas: Type D, characterised by cytoplasmic YAP/TAZ in 20–50% of tumour cells, and Type E, characterised by the absence of staining or weak cytoplasmic in less than 20% of the tumour (Fig. 3A). In the majority of prolactinomas (11 out of 16), YAP/TAZ staining was classified as Type E, where most of the tissue was negative for YAP and TAZ. In corticotropinomas, staining ranged from Type B to Type D (Supplementary Fig. 3A) and in somatotropinomas from Type B to Type E (Supplementary Fig. 3B). Results are summarised in Table 1. To determine if YAP/TAZ become elevated in aggressive/invasive tumours, we analysed their expression in one PRL-secreting carcinoma. This tumour appeared entirely negative for both proteins (Fig. 3B, Region 1) and only one area included cells with nuclear, but not cytoplasmic TAZ staining and cytoplasmic YAP immunoreactivity, similar to adult normal anterior pituitary (Fig. 3B, Region 2). Therefore, YAP/TAZ levels are low in differentiated tumours, and in the single malignant prolactinoma sample analysed. In summary, robust expression levels and more abundant nuclear localisation of YAP/TAZ are seen in non-secreting tumour types, harbouring a less differentiated cellular component, compared to tumours composed of differentiated cells.

### *Lats1* knockdown reduces *Gh* and *Prl* promoter activity in GH3 cells *in vitro*

**Figure 4:**
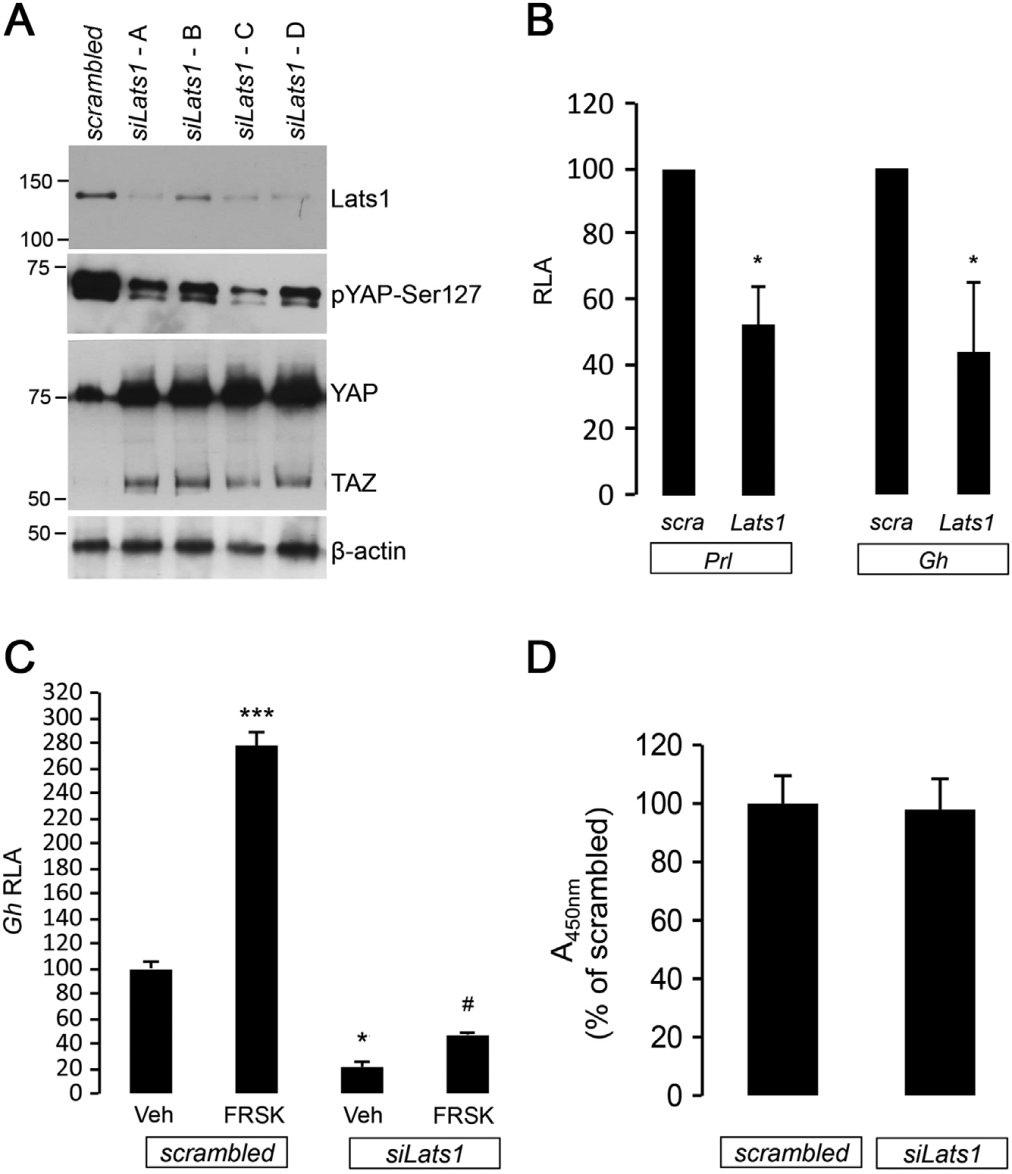
LATS1 inhibition with RNA interference suppresses the promoter activities of anterior pituitary hormones. (A) GH3 cells were transfected with a scrambled siRNA (control) or four different siRNA against rat *Lats1* for 48 h. Immunoblot shows the knockdown efficacy and the impact of decreased LATS1 protein on YAP phosphorylation at Ser127 and total YAP and TAZ protein levels. (B) Effect of *Lats1* knockdown on basal *Prl* and *Gh* promoter activity. Data are luciferase to β-galactosidase ratio, means ± standard deviation of three experiments (each in triplicate) presented as percentage of scrambled control. RLA, relative luciferase activity. *P < 0.05 to scrambled vehicle control. (C) Effect of *Lats1* knockdown on forskolin-induced (10 µM, 6 h treatment) *Gh* promoter activity. Data are means ± standard deviation of three triplicates and presented as percentage of scrambled vehicle control. *P < 0.05 and ***P < 0.001 vs scrambled vehicle control, ^#^*P* < 0.05 vs *siLats1* vehicle control. Veh, vehicle – the carrier in which forskolin was diluted. (D) *Lats1* knockdown does not affect cell proliferation (WST-1 colorimetric assay). Data are absorbance at OD450nm presented as percentage of scrambled siRNA control.

Our observations of a robust decrease or loss of YAP/TAZ in hormone-secreting pituitary tumours, compared to normal pituitary and non-secreting tumours, suggest a role for the Hippo kinases in promoting a hormone-secreting phenotype and/or repressing a progenitor/stem-like state. To this end, employing RNA interference, we inhibited LATS1, since it directly phosphorylates and marks YAP and TAZ for cytoplasmic retention and degradation. Knockdown of *Lats1* in rat mammosomatotropinoma GH3 cells reduced YAP phosphorylation at S127 and increased YAP and TAZ protein levels (Fig. 4A). This was accompanied by significant suppression of basal *Gh and Prl* promoter activity (Fig. 4B). *Lats1* inhibition also reduced activation of the *Gh* promoter following stimulation with forskolin compared to scrambled siRNA control (Fig. 4C). *Lats1* inhibition did not affect GH3 cell proliferation (Fig. 4D). These *in vitro* findings indicate that deregulation of the Hippo pathway may repress pituitary hormone synthesis and compromise the pituitary cell response to physiological hormonal stimuli.

## Discussion

Initially described in *Drosophila*, the Hippo pathway is now recognised as one of the most conserved molecular pathway in all metazoans, which is highly involved in fine-tuning of organ size through inhibition of proliferation and promotion of differentiation and cell death (Yu *et al.* 2015) with a contribution to tumourigenesis and cancer development (Mo *et al.* 2014). The present study demonstrates for the first time the expression patterns of two major downstream effectors of the Hippo pathway, YAP and TAZ, in the foetal and adult normal pituitary gland and provides evidence of Hippo pathway activity during embryonic development that persists into adulthood. Interestingly, the YAP/TAZ expression pattern is reminiscent of SOX2 in pituitary stem/progenitor cells recapitulating our previous findings in mice and indicating a potential link of the Hippo pathway to the stem/progenitor cell state (Lodge *et al.* 2016).

We investigated the expression of YAP/TAZ in three types of non-secreting/non-differentiated pituitary tumours: null-cell adenomas, ACPs and PCPs. YAP and TAZ were highly expressed in all ACP and PCP tumours. The expression pattern was nuclear and occasionally cytoplasmic in craniopharyngiomas that harbour known genetic mutations in *CTNNB1* or *BRAF* and was located to well-characterised tumour compartments known to express progenitor markers. ACP tumourigenesis is mainly driven by mutations in the *CTNNB1* gene that encodes for β-catenin, the central regulator of the WNT pathway (Sekine *et al.* 2002). Gain-of-function alleles of *BRAF* activate the RAS-RAF-MEK-ERK pathway and may drive tumourigenesis in PCPs, which appear almost exclusively in adults (Brastianos *et al.* 2014, Larkin *et al* 2014). In support of this, a mouse model overexpressing BRAF V600E in mouse pituitary precursors leads to increased proliferation of SOX2 stem cells and a block in differentiation (Haston *et al.* 2017). Interestingly, both pathways were described to crosstalk with the Hippo signalling cascade. YAP forms a transcriptional complex with β-catenin that is required for tumour transformation and survival (Rosenbluh *et al.* 2012). When core Hippo kinases are active, YAP/TAZ are degraded or sequestered in the cytosol where they limit WNT-β-catenin signalling (Varelas *et al.* 2010). In contrast, WNT activation induces YAP/TAZ translocation to the nucleus and target activation (Attisano & Wrana 2013, Azzolin *et al.* 2014). Similarly, RAS signalling activates YAP, and there is evidence for involvement of YAP in tumour resistance to pharmacological RAF-MEK inhibition (Reddy & Irvine 2013, Lin *et al.* 2015, You *et al.* 2015).

YAP and TAZ were also highly expressed in null-cell pituitary tumours, but at variable levels. Similar to craniopharyngiomas, they also often displayed strong nuclear staining. There is mounting evidence that TAZ and YAP nuclear localisation correlates with metastatic potential, low response to treatment and worse patient outcome in several solid tumours (Zanconato *et al.* 2016). The relatively small number of cases did not allow us to correlate the pattern of staining with histological or clinical data but also, recurrence may happen even after years of remission. Based on the recent WHO classification for pituitary adenomas, clinical aggressiveness is assessed by several clinical parameters such as tumour invasion (by MRI studies and/or intraoperative impression), in addition to mitotic count and Ki-67 index (Lopes 2017). Whether YAP/TAZ may directly promote an oncogenic phenotype in the pituitary through activation of genes involved in proliferation/survival/invasion directly or secondary to other genetic defects like the ones found in ACP and PCP is an intriguing possibility that remains to be explored.

In contrast to the majority of the non-differentiated tumours that often displayed strong nuclear staining, YAP/TAZ expression in the hormone-secreting prolactinomas was very low or completely absent, suggesting that in the human pituitary YAP/TAZ expression may be associated with a shift from high to low secretory potential and therefore a less differentiated state. Indeed, our *in vitro* model showed that increasing YAP and TAZ expression in lactosomatotroph GH3 cells, by knocking down their upstream regulator *Lats1*, dramatically reduces PRL as well as GH production without affecting cell proliferation. Interestingly in the case of GH, the effect is prominent also after cAMP/PKA stimulation with forskolin. These data reflect the observations in the *Lats1^−^^/^^−^* mice, which presented with low PRL and GH levels despite their hyperplastic pituitaries (St John *et al.* 1999). YAP/TAZ were shown to repress the differentiated state in other tissues (Lee *et al.* 2016, Yi *et al.* 2016, Cotton *et al.* 2017). Our data further support this notion and suggest that high levels of YAP/TAZ are associated with repression of hormone production and therefore a non-differentiated/progenitor state.

Treatment of pituitary tumours primarily relies on reducing hormone hypersecretion and its effects, decreasing the tumour mass and treating for any hormone deficiencies resulting from damage of normal pituitary tissue. Hippo signalling components and particularly YAP/TAZ have recently become attractive targets for new anti-cancer treatments (Johnson & Halder 2013, Nakatani *et al.* 2016). Our finding of increased expression of YAP/TAZ in non-secreting pituitary tumours reveals a previously unsuspected pathogenetic mechanism. Better understanding and targeting of the Hippo signalling cascade could introduce novel improved treatments for these intriguing and hard to manage tumours.

## Supplementary Material

Supporting Figure 1

Supporting Figure 2

Supporting Figure 3

## Declaration of interest

The authors declare that there is no conflict of interest that could be perceived as prejudicing the impartiality of the research reported.

## Funding

This work was supported by the Medical Research Council (MR/L016729/1) and a Lister Institute Research Prize to C L A. E J L, A S, S R B, M T and C L A were supported by the Deutsche Forschungsgemeinschaft (DFG) within the CRC/Transregio 205/1 (Project A06 to C L A and S R B and B17 to M T) and CRC/Transregio 127/2, as well as GRK 2251.

## Author contribution statement

Concept and design of experiments: P X, M T, C L A. Provision of samples: J M, J R A, A S, T S J, V P, R L, J F. Acquisition of data: P X, E J L, M T, J M, A S. Analysis and interpretation of data: P X, E J L, J M, M T, C L A. Writing and review of the manuscript: P X, E J L, M T, C L A. Supervision of the work: S A, S R B, M T, C L A.
