# Supplementary figures and images for: Non-secreting pituitary tumours characterised by enhanced expression of YAP/TAZ
Source: Endocr Relat Cancer. 2018 Aug 21;26(1):215–25. doi: 10.1530/ERC-18-0330 (PMC6215911; doi:10.1530/ERC-18-0330)

# a Fetal 14 weeks

H&E

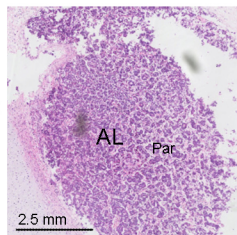

# Fetal 17 weeks

H&E

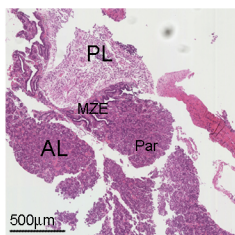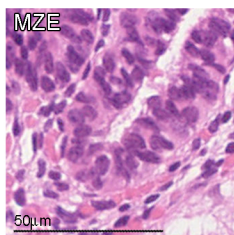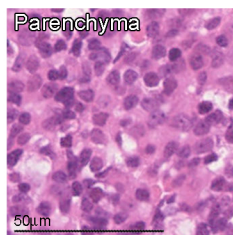

b

SOX2

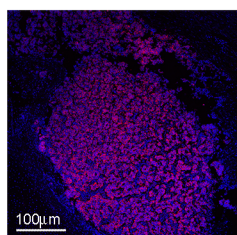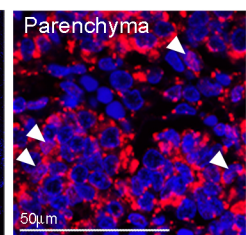

TAZ

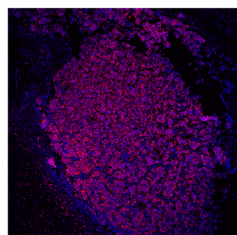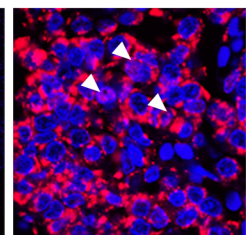

YAP

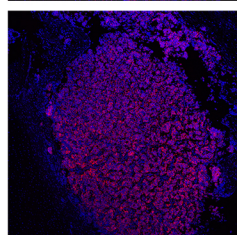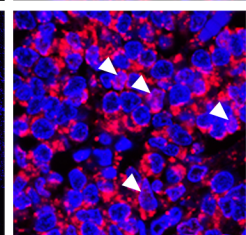

pYAP

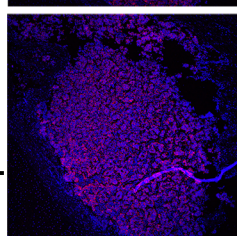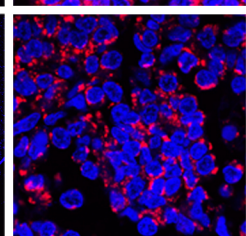

c

SOX2

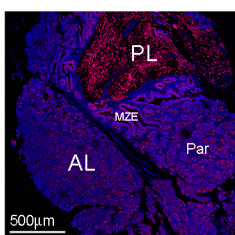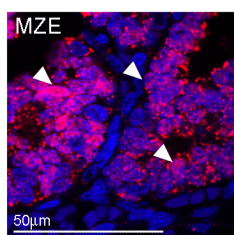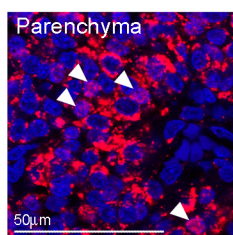

TAZ

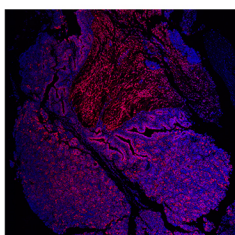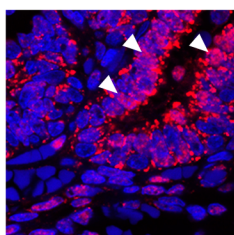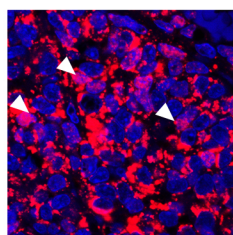

YAP

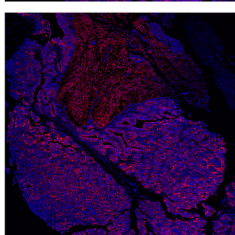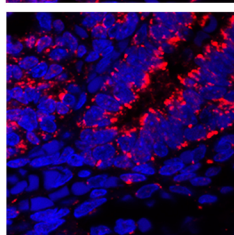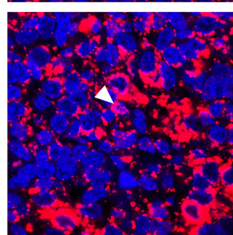

pYAP

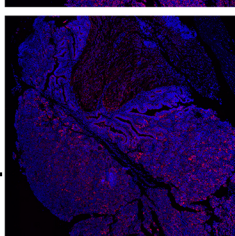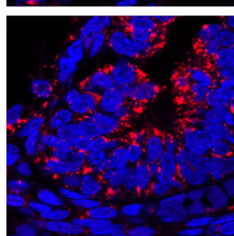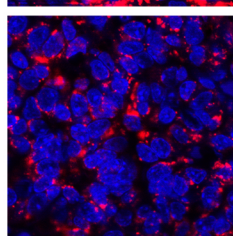

Supplement: Supporting Figure 1 [file erc-26-215-s001.pdf]
