# Supplementary material for: Non-secreting pituitary tumours characterised by enhanced expression of YAP/TAZ
Source: Endocr Relat Cancer. 2018 Aug 21;26(1):215–25. doi: 10.1530/ERC-18-0330 (PMC6215911; doi:10.1530/ERC-18-0330)
Supplement: Supporting Figure 2 [file erc-26-215-s002.pdf]

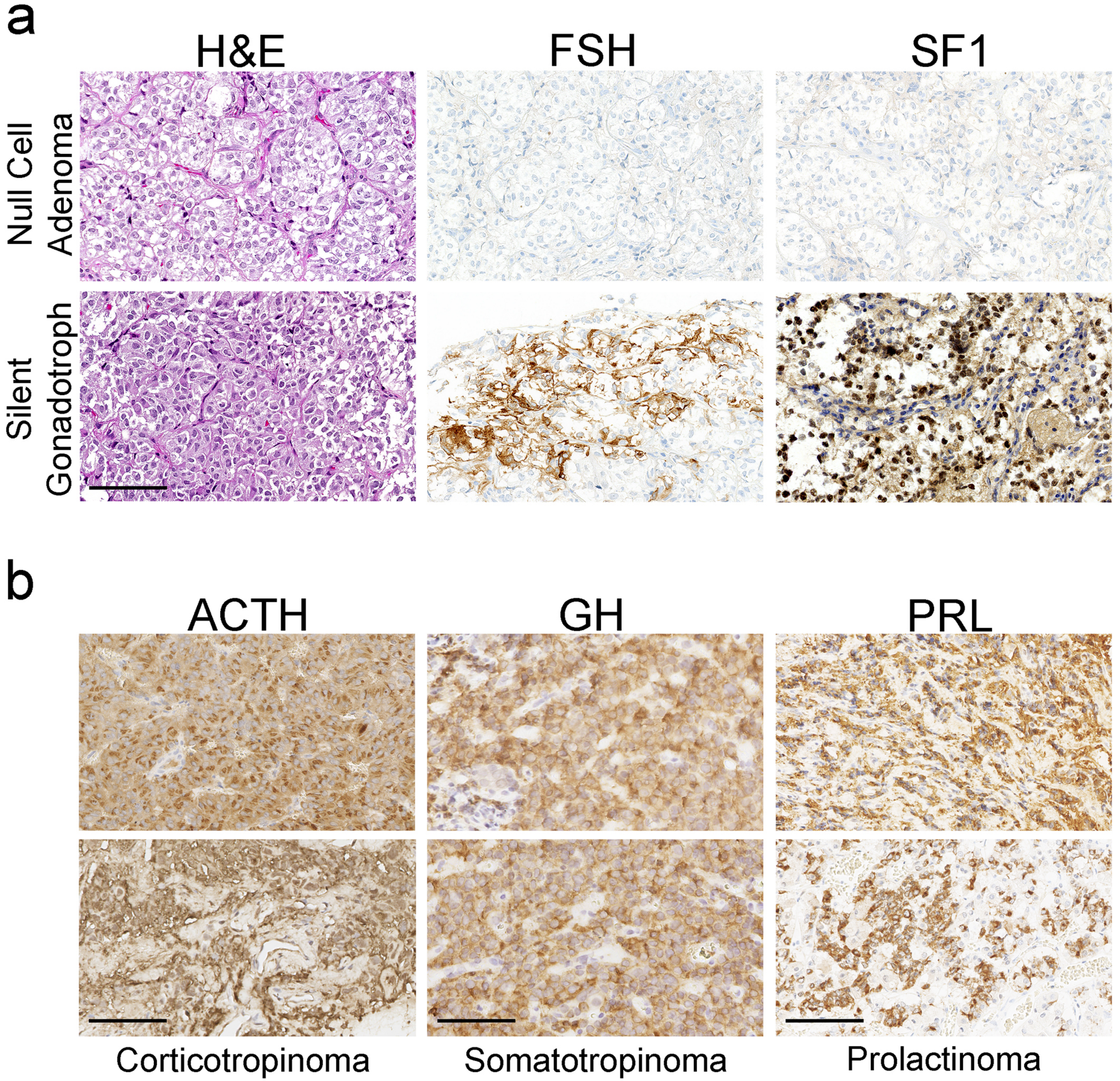
a
H&E
FSH
SF1
Null Cell Adenoma
Silent Gonadotroph
b
ACTH
GH
PRL
Corticotropinoma
Somatotropinoma
Prolactinoma
