# Supplementary material for: Non-secreting pituitary tumours characterised by enhanced expression of YAP/TAZ
Source: Endocr Relat Cancer. 2018 Aug 21;26(1):215–25. doi: 10.1530/ERC-18-0330 (PMC6215911; doi:10.1530/ERC-18-0330)
Supplement: Supporting Figure 3 [file erc-26-215-s003.pdf]

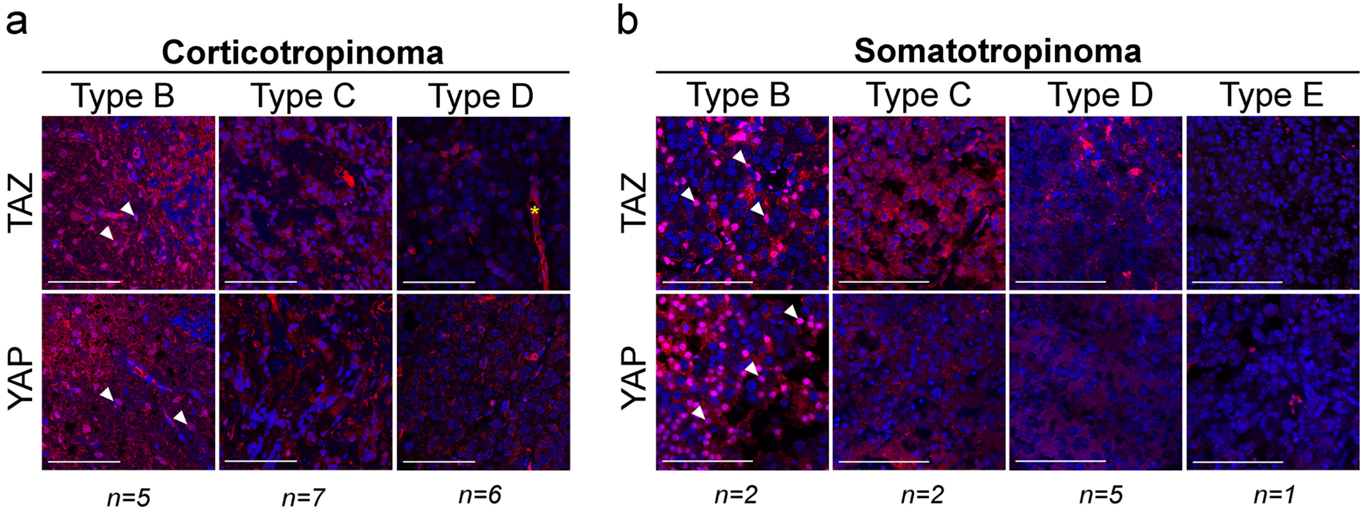
a
Corticotropinoma
Type B
Type C
Type D
TAZ
YAP
n=5
n=7
n=6
b
Somatotropinoma
Type B
Type C
Type D
Type E
TAZ
YAP
n=2
n=2
n=5
n=1
